# Comparison of veterinary drugs and veterinary homeopathy: part 1

**DOI:** 10.1136/vr.104278

**Published:** 2017-08-12

**Authors:** P. Lees, L. Pelligand, M. Whiting, D. Chambers, P-L. Toutain, M. L. Whitehead

**Affiliations:** 1 Royal Veterinary College, Hawkshead Campus, Hatfield, Hertfordshire AL9 7TA, UK; 2 Hall Manor, Kelly, Lifton, Devon PL16 0HQ, UK; 3 Toxalim, Ecole Nationale Veterinaire de Toulouse, France; 4 CertSAM, MRCVS, Chipping Norton Veterinary Hospital, Banbury Road, Chipping Norton, Oxon OX7 5SY, UK

## Abstract

For many years after its invention around 1796, homeopathy was widely used in people and later in animals. Over the intervening period (1796-2016) pharmacology emerged as a science from Materia Medica (medicinal materials) to become the mainstay of veterinary therapeutics. There remains today a much smaller, but significant, use of homeopathy by veterinary surgeons. Homeopathic products are sometimes administered when conventional drug therapies have not succeeded, but are also used as alternatives to scientifically based therapies and licensed products. The principles underlying the veterinary use of drug-based and homeopathic products are polar opposites; this provides the basis for comparison between them. This two-part review compares and contrasts the two treatment forms in respect of history, constituents, methods of preparation, known or postulated mechanisms underlying responses, the legal basis for use and scientific credibility in the 21st century. Part 1 begins with a consideration of why therapeutic products actually work or appear to do so.

We are all trying to understand our own age, and we rightly use the past to help us to do so. But we cannot gain this understanding unless we pay the past the respect it deserves. We must understand just how different it was (Moore 2010).

## Why medicinal products work or seem to work

European Union (EU) terminology refers to medicinal substance-based products. In this review these will be termed drug-based products. A drug may be defined as a medicine or other substance that has a physiological effect or acts on a pathophysiological process, when ingested or otherwise introduced into the body. For drug-based products, clinical use is based on established pharmacological actions and, in many cases, on established molecular mechanisms. In this review, such conventional medicinal products specifically exclude homeopathic products. A summary of the use of homeopathic products in animals in the EU has been provided by the European Council for Classical Homeopathy (2007). The EU definition (Directive 2001/83/EC, as amended) of a homeopathic medicinal product is ‘any medicinal product prepared from substances called homeopathic stocks in accordance with a homeopathic manufacturing procedure described by the European Pharmacopoeia or, in the absence thereof, by the pharmacopoeias currently used officially in the member states. A homeopathic medicinal product may contain a number of principles’. For homeopathic medicinal products, mechanisms of action are unknown (vide infra). Nevertheless, there are several possible explanations as to why and how products in both categories work or appear to work. They may possess genuine efficacy (something actually happens) or ‘apparent efficacy’ (something is only perceived to happen). In addition, there is also ‘indirect or vicarious efficacy’; for example, an owner wrongly perceives a behavioural problem in a dog and this triggers undesired behaviours. If treated, by a product of either class, the owner might then cease triggering the negative behaviour and the product, without direct action, receives credit for achieving a positive outcome.

### Coincidence

Commonly, there is an understandable but regrettable reluctance to accept that coincidence might be the explanation for a given observation. The fact that many illnesses resolve, irrespective of treatment given, means that resolution or improvement and treatment may simply be coincidental. If a veterinarian gives a treatment and the animal gets better, there is a strong cognitive bias (the post hoc ergo propter hoc bias [Rudolf 1938, Pinto 2001, Gay 2006]) to believe that the treatment is responsible, but this assumption might be misplaced.

Any cure can be confounded by many factors, which render establishing a causal relationship between treatment and cure difficult. Confounding factors may mask an actual association or, more commonly, falsely indicate an apparent association between treatment and outcome, when there is no actual association (Skelly and others 2012). For every effect, we commonly assume that there must be a specific cause, preferably the one favoured by each of us individually. Factors to be considered when assessing the efficacy of any product include: specific effects of the treatment, placebo effect, bias in observers’ assessment of patients’ response to treatment, the natural course of the disease, and effects of concurrent management of the illness, as discussed below.

### Specific effect of the treatment

If the treatment is actually effective, where efficacy may be underpinned by many preclinical studies and manifest in controlled clinical trials, that is called a specific effect. It is the active constituent(s) of the drug-based product or, for a homeopathic product, the unknown mechanism, which provides the claimed benefit. For a drug-based product, efficacy is achieved if a sufficient number of molecules reach and persist at the site of action (the biophase) for a sufficient period of time to act upon a biochemical/physiological pathway. Alternatively, a drug may act on some factor involved in a disease process; this would include a direct or indirect action on a parasite or microorganism present in or on the body. Beyond ‘working’ (or not), the degree of efficacy (ie, magnitude of response and the establishment of dose-effect relationships) is pivotal to the demonstration of efficacy for drug-based but not for homeopathic products.

### Placebo effect

Placebo effects are the main reason used by critics to explain apparent homeopathic effects, and are part of the ‘baseline’ to which the efficacy of any medication – conventional or homeopathic – is compared in randomised controlled trials (for example, Hektoen 2005, Shang and others 2005, Kayne 2006, Teixeira and others 2010, Brien and others 2011, Mathie and others 2012, Smith 2012, Vijayakumar 2012, Campbell 2013, Mathie and Clausen 2014). A placebo is a medical intervention that has a non-specific psychological or psychophysiological therapeutic effect and is thus lacking any known specific effect for the condition treated (McMillan 1999), but products with specific efficacy can also produce placebo effects. Placebo effects impact patients’ perception of their symptoms far more than they do the physiological and pathological processes of disease; any placebo effects on these more objective aspects of disease are typically small in magnitude and clinically irrelevant (Hróbjartsson and Gøtzsche 2010, Wechsler and others 2011). The basis of the placebo effect in people is experiencing a beneficial effect, arising from belief in the treatment, and based partly on confidence derived from consultations, leading to expectations on the part of the patient. In addition, there may be behavioural conditioning (Enck and others 2013). Mechanisms underlying the placebo effect are still poorly understood; they might be multiple and indeed might differ from circumstance to circumstance. A veterinary example is the display of separation-related behaviour in dogs, for which a conditioned placebo effect, suppressing signs of distress, was demonstrated (Sümegi and others 2014). It is clear that the placebo effect can and sometimes does operate for both homeopathic and drug therapies. Even if the mechanism(s) are obscure, the accepted view is that (in human medicine) a half to one-hour chat with a sympathetic and convincing homeopath can yield positive outcomes; all the collateral benefits of old-fashioned, reassuring, paternalistic medicine. This will be especially true where mind-over-matter considerations are pre-eminent to outcome. In Bavaria, it was reported that 88 per cent of GPs sent patients home with prescriptions for placebo drugs, the corresponding figure for the whole of Germany being 50 per cent (Jutte and others 2011, Kupferschmidt 2011).

In veterinary medicine, it is less easy to conceive if and how an animal can distinguish mentally between a homeopathic and drug-based product, if both are identical in presentation and similarly administered. For the huge majority of medical conditions, a placebo effect seems to be unlikely and counter intuitive, particularly as an animal cannot normally be expected to have the cognitive capacity to expect recovery or healing. The placebo component of the effect of a homeopathic veterinary product is presumably limited normally to the judgement of outcome, based on the subjective evaluation of the caregiver (veterinarian or animal owner) (Conzemius and Evans 2012, Talbot and others 2013, Gruen and others 2014, 2017). As in human medicine, a sympathetic veterinarian might provide the basis for placebo-induced benefit in the owner, for both drug-based and homeopathic products. The problem then is that the veterinarian and/or animal owner believes (wholly sincerely) that a beneficial response has occurred, but the animal may continue to suffer. Nevertheless, the potential beneficial effect of human contact on the health and physiological state of animals can be real (Mills and Cracknell 2013). In daily practice, this non-specific treatment effect may be especially important whereas, in a randomised controlled clinical trial, it will be randomly distributed between the treatment and control groups and of lesser importance in studies in animals than in people.

In so far as placebo effects occur in animals, for both drug-based and homeopathic products, explanatory theories have been based on: classical conditioning (as recognised in dogs responding to a saline injection as if it were morphine [Pavlov 1927]); cognitive expectancy; and release of endogenous opioids (McMillan 1999, Mills and Cracknell 2013). For further discussion on each of these aspects see Hektoen (2005). For in depth discussion on the placebo effect, see also Meissner and others (2011). In many instances, the placebo effect has been shown to work through recognised physiological/biochemical pathways and encompassing both central and peripheral nervous systems. Enck and others (2013) discuss physiological pathways in placebo analgesia, involving the descending pain modulatory network, and conditioned corticosteroid effects in patients with psoriasis.

### Bias in observers’ assessment of patients’ response to treatment

Doctors or veterinarians sometimes judge that a treatment has had an effect on a patient when, in fact, it has not. There are many examples in medical history of treatments that were thought to be beneficial, but were later proven to be ineffective or even harmful; well-known examples include blood-letting, use of anti-arrhythmics after ischaemic heart disease, hormone replacement therapy to prevent ischaemic heart disease in postmenopausal women, and radical mastectomy rather than more limited surgery for breast cancer (Prasad and Cifu 2015). Medical professionals are naturally inclined to believe that, if a patient improves after a treatment has been given, the improvement must have been a result of that treatment (post hoc ergo propter hoc bias). This is one example of many cognitive biases that can result in incorrect interpretation of the patient’s response to treatment (Rudolf 1938, Croskerry 2003, Gay 2006, Kahneman 2012, McKenzie 2014, Matute and others 2015, Canfield and others 2016).

### Other factors impacting on assessment of treatment efficacy

#### Non-specific healing effects

In addition to placebo effects and observers’ bias, other non-specific healing effects, regression to the mean (RTM) and the natural course of disease may all impact on efficacy – perceived or real. As discussed by Hektoen (2005), Mills and Cracknell (2013) and Talbot and others (2013), the elements potentially involved in the total effect of any treatment are: specific treatment effects statistically demonstrated in clinical trials; non-specific effects of treatment (such as the placebo effect); natural resolution of the signs of disease or deranged condition, including self-healing; RTM; concomitant support for treatments, such as nursing or reduced bodyweight; and combinations of these factors. RTM was first identified by Galton (1886) and has been discussed more recently by Morton and Torgerson (2003, 2005). In a well-designed randomised controlled trial (Lees and others 2017), all the factors listed, except the specific treatment effect, should be evenly distributed between treatment groups. Thus, the improvement in the placebo group is the sum of factors, such as non-specific treatment effects, natural history of the disease, RTM and the effects of concurrent nursing. These clearly must be non-specific effects, because no treatment with a specific effect was given to the placebo group. In the case of a veterinarian treating an individual patient, in many cases it is not possible to differentiate between non-specific effects and any specific effect of the treatment. For the individual clinical veterinarian treating the individual animal, all of these mechanisms may be operative, often resulting in treatments appearing to be effective when, in fact, they are not.

#### Concurrent management of patients

Many medical treatments are associated with additional changes in management of the patient, for example, nursing, rest, change of diet and treatment with other drugs. Many of these factors can lead to improvements in the disease that may be misattributed to the treatment being evaluated. For example, an obese dog given a medical treatment for osteoarthritis and also put on a weight loss diet may have reduced clinical signs, because of weight loss rather than the medical treatment.

#### The natural history of disease

Many diseases have a natural history, leading to mortality or morbidity or, more hopefully, partial or complete restoration of health. As Voltaire said, ‘the art of medicine consists in amusing the patient while nature cures the disease’. RTM comprises the natural fluctuation of variables around a mean, and its impact can be considered by way of example. A dog with osteoarthritis shows signs of reduced movement, joint stiffness and pain. The owner seeks veterinary advice, a medication is prescribed and the dog shows improvement. If, even in the absence of treatment, the signs wax and wane (as may well occur in the osteoarthritic dog), the owner and veterinarian understandably, but in part or in whole wrongly, may attribute the improvement to the administered product. Talbot and others (2013) discussed this problem in relation to a feed supplement used in head-shaking horses, a condition well known for its intermittency. RTM may occur in an individual animal (as in the example cited above) or as a group phenomenon and in both cases the observed increase or decrease may be mistakenly attributed to a specific treatment effect (Morton and Torgerson 2005).

#### The body’s natural healing mechanisms (and their interaction with efficacious medicines)

The natural defence mechanisms of the body in microbial and other diseases can prove highly effective in providing a clinical cure or, better still, a microbiological cure (the gold standard). In microbial disease, the administered drug acts in concert with many immune-based mechanisms, notably the scavenging action of white blood cells, working to defeat the invading pathogen. Drusano and others (2010) calculated that, if antimicrobial therapy drives the bacterial (*Staphylococcus aureus*) population down to between 10^2^ and 10^3^ colony forming units/g, it is highly likely that the residual population will be eradicated by the immune system and, moreover, will be achieved with minimal amplification of resistant mutants.

In veterinary medicine, the use of antimicrobial drugs in prophylaxis (now under challenge within the EU) is deemed to give the immune-based pathways invaluable support. In metaphylactic use (sometimes referred to as mass medication) drugs are administered collectively to animals, in which the bacterial population exceeds the capacity of the natural defences of the animals to work without support. In therapy, especially in the presence of immune deficiencies and heavy bacterial loads, the prudent use of antimicrobial drugs in animals is essential to welfare through restoration of health. Their actions may be attributable to: direct killing, reduced pathogen pathogenicity or the enhancement of host immune pathways.

With other deviations from normal ranges, the body has the ability, through biochemical, physiological and endocrinological pathways, to restore systems to normal – the homeostasis of the body. These systems are finely balanced and usually integrated, so that, for example, there is a tonic influence of sympathetic nerves to arterioles to keep them in a state of partial constriction. The same arterioles are under an opposing tonic vasodilator effect of the nitric oxide system. The system can fail, arterial blood pressure may rise and the resulting hypertension may require the attention of a suitable drug. Thus, the homeostatic pathways may be suboptimal in a hypertensive cat, but they are most likely to be still operational and the pharmacological agent may play only a minor but essential role in assisting the body to restore homeostatic balance.

Likewise, there are innumerable integrated systems, keeping within normal ranges blood glucose and blood cell counts. Drugs which act on neural, physiological and endocrinological pathways are generally working in concert with the body’s enzymes, neurotransmitters and hormones and, even in the presence of a drug or homeopathically energised water, it may be that it is the homeostasis which plays the dominant and even the sole role. There will be many other circumstances, when the drug is required not to work in concert with but to combat a deranged physiological system; if sympathetic nervous vasoconstrictor tone to arterioles is increased, the drug is needed to correct that. Many other drugs are used to counter natural physiological processes, such as anaesthetics, while others suppress a natural and useful but unwelcome process, such as inflammation.

In summary, placebo effects are those beneficial effects arising from use of a treatment that are not due to the properties of the treatment itself, and therefore must arise from cognitive processes such as belief and expectation. However, placebo effects are only one of many non-specific factors that can give rise to an improvement from treatment. As discussed above, other non-specific effects, that do not arise from the treatment at all, include RTM, other coincidental improvements, and effects of concurrent nursing or change of diet. Additional factors can cause perceived but not real improvement, for example, observer bias and selection bias. All of these non-specific effects may occur together, and between them give rise to the improvement seen in the placebo-control group in a randomised controlled trial; that is, improvement that is not due to the specific effect of the treatment. Because all of these non-specific effects occur in the placebo-control group, they are sometimes referred to as ‘placebo effects’ although strictly, this is an error of terminology because true placebo effects are only one contributor to the totality of non-specific effects. In animals, with far less ability to experience beliefs and expectations about the healing effects of treatments, true placebo effects will contribute much less to the non-specific effects than in people.

## History

### Homeopathy

The history of homeopathy has been covered elsewhere (Bellavite and others 2005, Kayne 2006, Loudon 2006, Cook 2008, Campbell 2013). Briefly, the fundamental principle of homeopathy, that ‘like cures like’, was proposed, in 1796, by Samuel Hahnemann (1755-1843), as an alternative to other therapies then in use; primarily herbalism, bleeding, purging, emesis, blistering and sweating (Porter 1997, Wootton 2006). By 1814, Hahnemann was using highly-diluted homeopathic remedies similar to those used by homeopaths today (Hahnemann 1814). Before inventing homeopathy, Hahnemann qualified as a doctor and worked as a conventional physician, then as a translator of scientific articles and a writer. He also studied chemistry. He translated a conventional Materia Medica (by William Cullen, 1710-1790) into his native German and found it to be lacking. In its place, he devised and advocated the principles of homeopathy.

Homeopathic remedies are based on three central tenets, ‘The Law of Similars’ (similia similibus curantur), ‘The Law of Infinitesimals’ and ‘The Law of Succussion’, each arising from the writings of Hahnemann, in particular his ‘Organon of Medicine’ (Hahnemann 2002). According to The Law of Similars, signs and symptoms can be cured by substances that can cause those signs and symptoms in healthy individuals (Hahneman 2002, Kayne 2006, Owen 2015). The naming of homeopathic products is usually in Latin. Remedies are listed in homeopathic Materia Medica (Hahnemann 1814, Boericke 2008, several others available at various Internet sites [International Academy of Classical Homeopathy 2016]), together with the signs and symptoms the remedy is thought to be effective for (Lilley 2008). Homeopaths also use repertories, which list signs and symptoms, and for each give the remedies thought to be effective for that sign or symptom (Boericke 2008). For example, insomnia can be treated by the coffee bean remedy, Coffea cruda (Boericke 2008) – coffee contains the central nervous system stimulants caffeine and theophylline – or a common cold can be treated by the onion remedy Allium cepa (Boericke 2008) – onions make the eyes water. For Hahnemann, as for conventional medical doctors in the late 18th century, working before the advent of science and modern medicine, the human body was a black box; a medicine goes in and the effects (any change in symptoms) come out, there being no knowledge of or much interest in ‘the in between’. How the products of either category worked was unknown and inconsequential.

Various forms of like-cures-like concept were present in medical writings long predating Hahnemann, for example, Hippocrates in the 4th century BC and Paracelsus in the 16th century (Kayne 2006) and the general concept was present among medics in the late 18th century. The Reverend Edward Stone of Chipping Norton described in 1795 (one year ahead of Hahnemann) the treatment of agues by the willow (bark and leaves) noting, ‘as this tree delights in a moist or wet soil, where agues (fever) chiefly abound the general maxim that many natural maladies carry their cures along with them or that their remedies lie not far from their causes was so very apposite to this particular case that I could not help applying it’ (Wood 2015). We now know that, in this case, there is a conventional pharmacological explanation; the willow contains the glycoside salicin, which has anti-inflammatory and antipyretic effects. With advances in chemistry, this led in 1865 to the first synthetic analgesic drug of the non-steroidal anti-inflammatory (NSAID) class, salicylate; this then led in 1895 to acetylated salicylate, aspirin, followed by a plethora of drugs of the NSAID category. However, as a general principle, the like-cures-like concept is arbitrary and has no general credibility, notwithstanding its apparent but superficial symmetry. The general concept of ‘like-cures-like’ has been practised by many cultures over the millennia (Fraser 1922).

Stone’s ‘like-cures-like’ is of a qualitatively different type to that of homeopathy. In the Stone example the property of the substance used to treat a disease that is ‘like’ the disease is some observable physical attribute of the substance: in the case of the willow, it grows in damp places, and – in the thinking of the time – diseases tend to occur in damp places. This is a different ‘like-cures-like’ concept to homeopathy, in which the property of the substance used to treat a disease that is ‘like’ the disease is the ‘symptom picture’ induced in healthy volunteers by ingestion of the substance (in the early years of homeopathy) or by ingestion of a remedy made from the substance (for much of the history of homeopathy).

The fundamental principle of homeopathy is that something that induces specific signs and symptoms will also cure the same signs and symptoms. For veterinary medicine, we should note that animals do not have symptoms; symptoms are what humans report (headache, bellyache, disorientation), while signs are what we can observe and sometimes measure (rise in body temperature, tachycardia). Therefore, humans can have both symptoms and signs and non-human animals show only signs; the symptoms are known only to the individual animals.

Hahnemann’s second law, the Law of Infinitesimals challenges the scientifically based principles of biochemistry, physiology, endocrinology and pharmacology, of more molecules producing greater responses; the classical concentration/dose-response relationships (see part 2 of this review; Lees and others 2017). In complete contrast, Hahnemann’s second Law states that greater responses are achieved with less, over a huge range of dilutions. With repeated dilutions in (usually) water or alcohol, potency increases. A starting solution (called the ‘mother tincture’) of the ‘active’ is diluted either 1:10 (decimal) or 1:100 (centesimal), then that diluted solution is again diluted by the same degree, and the process continued (Kayne 2006, 2008). The degree of dilution of a remedy is referred to as its ‘potency’ – a 6c potency remedy has been diluted 1:100 six times (therefore, 10^-12^ dilution) and an 8d potency remedy has been diluted 1:10 eight times (10^-8^ dilution). Homeopathic products are provided over a wide range of ‘potencies’; in the UK 6c, 12c, 30c and 200c seem to be the most commonly used, but homeopaths’ preference varies from country to country (Kayne 2006). Most over-the-counter homeopathic remedies are 30c.

The number of molecules of the ‘active’ agent decreases rapidly with dilution and, as implied by Avogadro’s number (6 x 10^23^) beyond 12c (a dilution of 1x10^-24^) there is unlikely to be even one molecule of the starting substance present in the remedy (Vickers and Zollman 1999). At 12c dilution of a mole of starting substance, there is a 60.2 per cent chance of one molecule remaining. At 30c (10^-60^ dilution), to have one molecule of ‘active’ remaining would require a mass of water molecules of 2.99x10^34^ kg, more than 15,000 times the mass of the Sun of 1.99x10^30^ kg (Grimes 2012). It is estimated that there are approximately 10^80^ particles in our universe – 10^80^ corresponds to 40c dilution.

Succussion is the basis of the third Law. It is a specific type of vigorous shaking or tapping at each dilutional stage (Kayne 2006, 2008); this agitation is believed to ‘potentise’ or ‘dynamise’ the remedy, and is what causes the claimed healing power to not only pass from the less diluted stage to the more diluted stage, but to become more potent as it does so. Hahnemann believed that he had made a breakthrough discovery, while transporting his products in a horse drawn carriage. On the basis of uncontrolled observations, he judged that the vigorous shaking this involved increased the potency of his remedies even further beyond the dilution effect. Another equine contribution to homeopathy came in the form of his bespoke striking board used for succussion, constructed by a saddle maker, with leather on one side and stuffed with horsehair.

The preparation of homeopathic products today, as historically, involves shaking or tapping at each dilutional stage. A usual procedure is to strike the container between 10 and 50 times against an elastic object. According to Peter Fisher’s (homeopath and Clinical Director and Director of Research at the Royal London Hospital for Integrative Medicine) evidence to the UK House of Commons Science and Technology Committee (2010) ‘you have to shake it vigorously…. if you just stir it gently, it does not work’. Repeated dilution and succussion achieves ‘potentisation’ such that the healing power – the unidentified curative property – imparted to the remedy by the starting substance is retained (indeed increased with each shaking) by the water molecules. As Hahnemann wrote, the shaking procedure releases ‘dynamic forces from the diluents which were preserved and intensified with subsequent dilutions’. The nature of these ‘dynamic forces’ is not known; like Hahnemann (2002) himself, many contemporary homeopaths refer to them using terms such as ‘vital force’ or ‘life energy’, as used in homeopathy texts (Kayne 2006, Nicolai 2008, Owen 2015), and apparent from internet searches for these terms with ‘homeopathy’. These terms emphasise the mystical, vitalist nature of the belief system underlying homeopathic practice. The mechanisms by which homeopathic remedies effect improvements in signs or symptoms is not known, but homeopaths often refer to their remedies ‘balancing’ unspecified ‘energies’ in the body, or correcting a disturbance of the body’s ‘vital force’ (Bell and others 2004, Kayne 2006). However, the nature of these energies is likewise not known and their existence is unproven. They appear not to be detectable grossly, for example, by sight or touch, or by radiography, scintigraphy, ultrasound or CT or MRI scans. All three laws of homeopathy – similars, infinitesimals and succussion – are arbitrary, having been invented by Hahnemann, but never demonstrated to have a physical basis. Homeopaths often speculate that modern scientific concepts such as electromagnetism or quantum effects (Kayne 2006) might underlie the claimed efficacy of their remedies, and frequently refer to the ‘vital force’ and the action of their remedies in terms of ‘vibrations’ and ‘resonances’ (Kayne 2006). Thus, homeopathy is pseudoscientific.

### Pharmacology

The history of pharmacology spans less than 200 years. It derived from Materia Medica, which was practised for at least two millennia up to the late 19th/early 20th centuries. Early practitioners were Hippocrates and Galen. In the first known pharmacopoeia, the physician Pedanius Dioscorides wrote, in the first century BC, ‘the leaves of the willow being beaten small and drank with a little pepper and wine do help such as are troubled with the Iliaco Passio (colic). The decoction of the leaves and bark is an excellent fomentation for the gout.’ His De Materia Medica, was in continual use for more than 1500 years.

Writing around the time of Hahnemann (mid 18th century) Voltaire described pharmacology as ‘the pouring of drugs of which one knows nothing into a patient of whom one knows less’.

## Evolution of thinking 1796-2016

In 1796, the year of revelation to Hahnemann, there was available for use in both human and to a lesser degree veterinary medicine, Materia Medica (the use of plant parts or their extracts), blistering, bleeding, purging, sweating and emesis as the main bases for treatment, together with surgery, which in many cases was savage butchery. The skilled surgeon’s greatest asset was speed rather than quality. Medical treatment was largely based on the concept of balancing the four humours, and bloodletting was the primary treatment (Porter 1997, Wootton 2006).

Human doctors not only practised but prided themselves in these procedures. The period from 1780 to 1850 has been described as the time of ‘heroic medicine’. A popular ditty of the day was penned by John Coakley Lettsome (1744-1815) founder and President of the Medical Society of London ‘I, John Lettsome, blisters, bleeds and sweats ‘em; if, after that, they please to die; I John Lettsome’ (Scott and Scott 2008). Perhaps the initial success of homeopathy was due to the fact that it obeyed Hippocrates’ first principle of treatment: above all do no harm, giving it, over the conventional medicine of the time, a better risk:benefit ratio.

If the reaction of Hahnemann to these medical practices was derision or despair, one can only, with the benefit of hindsight, sympathise. Now, these barbaric procedures have been swept away by curiosity, observation, trial and error, experiment and serendipity (the bases of the scientific method), facilitated by the advances in knowledge, first of chemistry, then biochemistry/physiology, then cell and molecular biology, all dependent on increasingly sophisticated measuring and analytical techniques. It is true that throughout the 19th century quacks continued to peddle quack medicines, but the ascendency of the scientific method had largely put paid to the practice of quackery by doctors by the first quarter of the 20th century, as opposed to the practice of quackery by non-medical people, which continues.

On the veterinary scene, James White (1816) of Exeter, was way ahead of his time when he wrote; ‘within these few years only, has the veterinary art acquired a distinct appellation, and a solid foundation in this country. Receipts handed down by traditionary skill, in which ingredients were accumulated without judgment or discrimination, constituted the principles and practice of what was termed Farriery… It is only since the institution of the [London] Veterinary College, that the anatomy and physiology of the horse have been properly investigated, and the effects of medicines on his body correctly ascertained, by numerous and appropriate experiments, both in health and disease; so that a secure foundation is now laid; and, as long as scientific men continue to study and practise the veterinary art, it must necessarily be in a progressive state of improvement’.

The quack medicines of earlier centuries were largely based on spurious or unsubstantiated Materia Medica products. Now, almost nothing remains in 21st century therapeutics, except for some fine examples of the active constituents of Materia Medica remedies; we have quinidine, quinine, morphine, atropine, digitalis glycosides, d-tubocurarine and, derived from the willow, salicylate and its successors. We still have major therapeutic uses for the extracted chemicals of plants, but as drugs in 99 per cent plus purity form. Now, therefore, we have better control of the dose, a lesser likelihood of overdose and less opportunity for unwanted effects from the other constituents/adulterants of the plants or their extracts. And, of course, we have over the last 75 years, the example of the magic bullets (penicillin, streptomycin, tetracycline and their derivatives and successors) isolated from soil dwelling microorganisms or produced semi-synthetically or synthetically in the laboratory.

The steady development of conventional therapeutics has been an ongoing, often unplanned process, proceeding by an incremental, bottom up evolution. It began with the ideas of the Enlightenment. Charles Darwin, Claude Bernard (an early advocate of evidence-based medicine [Morabia 2006]), Louis Pasteur and Robert Koch were children of The Enlightenment and we are its great, great grandchildren. Johnson (2010) has written that both biological and technological developments comprise a ‘gradual but relentless probing of the adjacent possible, each new innovation opening up new paths to explore’. As the scientific method was refined, and new technologies developed, more was learned about chemistry, biology, physiology, biochemistry, microbiology and pathology, allowing the rational development of treatments. Moreover, in the 20th century medical science developed the randomised controlled clinical trial, allowing the objective testing of novel treatments.

In contrast, homeopathy was invented by one man, living at a time of minimal scientific understanding of biology and pathology. It has remained essentially unchanged. While there may now be many more homeopathic remedies, the underlying concepts and philosophy, and the methods of preparation (huge dilutions, succussion, etc), are essentially the same; the Laws are inviolate. An assumption underlying homeopathy is that disease signs are an expression of a disturbed vital force, affecting the whole organism and the treatment is intended to restore the ‘energetic balance’ of the individual (Bell and others 2004, Kayne 2006, Nicolai 2008, Owen 2015). The actual mechanisms remain obscure, implausible for most people and incompatible with scientific knowledge accumulated over the last two centuries.

The belief system of homeopaths is vitalist in that it posits that the phenomena of life are dependent on a force or principle distinct from purely chemical or physical forces – there is something ‘special’ about living tissue, above and beyond its content of atoms and molecules. Vitalism is a discredited scientific hypothesis that Ridley (2015) describes as a superstition in headlong retreat. Vitalism underlies most traditional healing practices, and the Hippocratic ‘four humours’ tradition that dominated Western medicine until disproven by modern science. Vitalism has been diminished by the advances in pharmacological, biochemical, cellular and molecular biologies, not least by the discovery that ‘the secret of life’ – DNA – turned out to be an infinitely combinatorial message, written in three-letter words in a four-letter alphabet. This discovery is inconsistent with the concept of a ‘vital force’. From psychology, superstitious adults tend to explain biological processes in terms of vitalist causality and energy transmission, and such conceptual confusions are associated with belief in alternative medicine (Lindeman and Saher 2007), which is itself associated with intuitive rather than rational thinking styles (Saher and Lindeman 2005) and belief in other supernatural and paranormal phenomena (Grimmer and White 1990, Saher and Lindeman 2005).

In the words of Hahnemann, diseases ‘are solely spirit-like (dynamic) derangements of the spirit-like power (the vital principle) that animates the human body’. We put the question, does a spirit-like power animate animal bodies too? Contemporary homeopaths still refer to spiritual aspects along with ‘life energy’ or ‘vital force’ when discussing the actions of their remedies (Kayne 2006). It is clear that the gulf between homeopaths and the great majority of human and animal doctors is not simply one of how to compare using common standards (McKenzie 2012). It is a gulf of mind-set, between a proven or plausible mechanism of action of the latter, and the mystical, superstitious beliefs of the former.

While homeopaths are vitalists, their belief system spreads more widely. Homeopathic practice implies the belief that there is some property – an ‘essence’ – in each of the substances or objects they make their remedies from; it is that essence which gives rise, via potentisation (dilution, succussion, etc), to the specific curative properties of the remedy. There are thousands of remedies, each with specific properties; that is, they treat only certain signs or symptoms or patients and not others, and seemingly no limit to what substances or objects remedies can be made from (vide infra). Hence, presumably every substance or object contains an essence. The belief that inanimate substances and objects, as well as animate objects such as plants and animals, have an essence (especially if that is construed as a ‘vital force’) places homeopathy in the mystical tradition of animism – the belief in a supernatural power that pervades, and can influence, the material universe. Moreover, the essence is beneficial for humans – indeed, potentising remedies for the treatment of ill humans and animals seems to be the only identified function or use for the essence. Hence, homeopathic beliefs are also ‘anthropocentric’ – believing that the universe, with this essence existing in every substance or object, exists as it does for the benefit of humans. These vitalistic, animistic, anthropocentric beliefs are part of the mystical belief systems universal to human cultures thoughout history.

## Constituents

### Homeopathic products

Contemporary homeopaths follow Hahnemann’s example of listing, in Materia Medica, their remedies together with the ‘symptom picture’ for each and dosage information (Lilley 2008). The symptom picture is established primarily by means of ‘provings’ or ‘pathogenetic trials’ (vide infra) and partly by observations of clinical responses to a remedy, and indicates which signs or symptoms the remedy can be used to treat in a patient (Belon 1995, Kayne 2006, Campbell 2013, Sherr 2015). For homeopathic products in humans, the proving involves a group of several volunteers or just one person. Each imbibes a number of doses of the remedy being ‘proved’, with contemporary provings typically using remedies diluted beyond the Avogardro limit. Each volunteer keeps a diary of the physical and emotional sensations experienced. On completion of the proving, the ‘master prover’ collates information from the diaries and this becomes the ‘symptom picture’ for that remedy and is recorded for homeopaths to reference in practice (Kayne 2006, Riley 2008, Campbell 2013, Sherr 2015). The scientific basis of homeopathic provings is not established. Furthermore, for veterinary products obvious practicalities dictate that these procedures cannot be followed when the recipient is an animal.

The components of homeopathic products are water (in some cases alcohol also), dissolved gases, impurities (a variety of inorganic and organic molecules of unknown amounts), and variable amounts of the ‘active’ agent, dependent on the degree of dilution, but less than one molecule at the high dilutions commonly used in practice and supplied as over-the-counter remedies (Kayne 2006). ‘Nanoparticles’ of the starting material have been demonstrated in some commercially available 30c and 200c remedies made from metals in India (Chikramane and others 2010), presumably due to imperfect dilution, or contamination after dilution, during preparation. There are thousands of remedies in published homeopathic Materia Medica (Boericke 2008) and available via the internet, with frequent new remedies being homeopathically ‘proved’ and used in practice (Kayne 2006, Riley 2008, Sherr 2015). There appears to be no restriction on what can be used as an ‘active’ to create a remedy; ‘actives’ include viruses, bacteria, animals, plants, minerals, chemicals, conventional drugs, man-made objects, and physical radiations and energy fields (the last two referred to as ‘imponderables’ by Hahnemann and modern homeopaths). Examples include: honey bee (Apis mel), emperor dragonfly (Anax imperator), duck offal (Oscillococcinum), green iguana (Iguana iguana), human placenta (Placenta humanum [Welsh]), Kentucky bluegrass (Poa pratensis), lava (Hekla lava), gunpowder (Carbon-sulphur-kali-nitricum), permethrin, condom (Latex vulcani), the Berlin Wall (Murus Berlinensis), Hadrian’s Wall (Vallum Aelium), car exhaust fumes, electricity (Electricitas), magnetic field (Magnetis poli ambo), emanations from televisions, X-rays (X-ray), and light from the planet Venus (Venus stella errans) – all of which can be found listed in homeopathic Material Medica or as homeopathic provings on the internet, and can be purchased from homeopathic pharmacies (www.helios.co.uk). Some homeopathic products contain sugar, but this is not claimed to be essential to efficacy (except in the homeopathic remedy Saccharum officinale, prepared from pure cane sugar as the ‘active’). Each remedy is claimed to possess specific healing properties, which can be used to treat only certain signs or symptoms, but not others, or only patients with certain characteristics, but not others; yet homeopaths appear to believe that all remedies exert their effects via a single (unknown) process (Kayne 2006, Nicolai 2008).

Remedies may be dispensed in liquid form, but can also be mixed with or dropped or sprayed on to other pharmaceutical preparations to create homeopathic creams, ointments, pills and powders (Kayne 2006, 2008). Once formulated, there are minimal costs to marketing, only extremely limited regulatory requirements to be negotiated, with no comparisons with other products, homeopathic or otherwise, required. Regulatory authorities recognise that the products are lacking in ingredients with specific actions and it is assumed that no toxicity will arise in the absence of actives. Therefore, it is further assumed that there can be no residues in edible tissues of food producing species and hence no meat/milk withholding periods are required.

### Drug-based products

For each drug-based product, there must, by definition, be one or more actives. However, it is rare for drugs to be marketed as the drug substance alone. Almost invariably they are formulated, for oral, parenteral or local administration, as solutions, suspensions, tablets or capsules, which contain other compounds, the excipients. Generally, no therapeutic activity is claimed for the excipients, but they are essential to ensure such properties as sterility and syringability and as bulking or flavouring agents. While themselves not active on biological systems, excipients can markedly influence pharmacological and therapeutic outcomes. This occurs principally by affecting the rate and extent of absorption of the active constituents.

Each active in conventional drugs is perceived to have a specific chemical, biochemical or physiological mechanism of action by which it brings about its clinical effects, and sometimes other mechanisms of action by which adverse effects arise. For many drugs the mechanism of action is proven, and for most drugs without proven mechanisms of action, scientifically plausible mechanisms exist. For discussion of the bases of efficacy of constituents of homeopathic and drug-based products, and the evidence regarding their clinical efficacy, see part 2 of this review (Lees and others 2017).

## Conflict of interest statement

None of the authors of this article has a financial or personal relationship with other people or organisations that could inappropriately influence or bias the content of the paper. D. Chambers and M. Whitehead are members of the Campaign for Rational Veterinary Medicine, an informal group of veterinary surgeons countering the promotion and use of implausible and irrational therapies by veterinary professionals.
